# Beyond Breast Cancer: Mammographic Features and Mortality Risk in a Population of Healthy Women

**DOI:** 10.1371/journal.pone.0078722

**Published:** 2013-10-25

**Authors:** Rachel A. Murphy, Catherine Schairer, Gretchen L. Gierach, Celia Byrne, Mark E. Sherman, Thomas C. Register, Jingzhong Ding, Stephen B. Kritchevsky, Tamara B. Harris

**Affiliations:** 1 Laboratory of Epidemiology, and Population Sciences, National Institute on Aging, Bethesda, Maryland, United States of America; 2 Division of Cancer Epidemiology and Genetics, National Cancer Institute, Bethesda, Maryland, United States of America; 3 Department of Preventive Medicine and Biometrics, Uniformed Services University of Health Sciences, Bethesda, Maryland, United States of America; 4 Sticht Center on Aging, Section on Comparative Medicine Pathology, Radiology, Winston-Salem, North Carolina, United States of America; 5 Sticht Center on Aging Section on Gerontology and Geriatrics, Wake Forest School of Medicine, Winston-Salem, North Carolina, United States of America; The Institute of Cancer Research, United Kingdom

## Abstract

**Background:**

Breast fibroglandular (dense) tissue is a risk factor for breast cancer. Beyond breast cancer, little is known regarding the prognostic significance of mammographic features.

**Methods:**

We evaluated relationships between nondense (fatty) breast area and dense area with all-cause mortality in 4,245 initially healthy women from the Breast Cancer Detection Demonstration Project; 1,361 died during a mean follow-up of 28.2 years. Dense area and total breast area were assessed using planimeter measurements from screening mammograms. Percent density reflects dense area relative to breast area and nondense area was calculated as the difference between total breast area and dense area. Hazard ratios (HRs) and 95% confidence intervals (CIs) were estimated by Cox proportional hazards regression.

**Results:**

In age-adjusted models, greater nondense and total breast area were associated with increased risk of death (HR 1.17, 95% CI 1.10-1.24 and HR 1.13, 95% CI 1.06-1.19, per SD difference) while greater dense area and percent density were associated with lower risk of death (HR 0.91, 95% CI 0.86-0.95 and HR 0.87, 95% CI 0.83-0.92, per SD difference). Associations were not attenuated with adjustment for race, education, mammogram type (x-ray or xerogram), smoking status, diabetes and heart disease. With additional adjustment for body mass index, associations were diminished for all features but remained statistically significant for dense area (HR 0.94, 95% CI 0.89-0.99, per SD difference) and percent density (HR 0.93, 95% CI 0.87-0.98, per SD difference).

**Conclusions:**

These data indicate that dense area and percent density may relate to survival in healthy women and suggest the potential utility of mammograms beyond prediction of breast cancer risk.

## Introduction

Tissue density, a reflection of the physical and biochemical composition of the tissue, can be estimated from the Hounsfield Unit of computed tomography images. Tissue density is particularly useful for capturing a variety of obesity-related health risks as the Hounsfield Unit provides an indication of adipose infiltration into tissue. For example, low liver density reflects fat accumulation, a risk factor for insulin resistance and type 2 diabetes [1,2]. Low skeletal muscle density is also associated with insulin resistance and type 2 diabetes [3,4] as well as increased mortality risk [5], fracture [6] and mobility limitation in old age [7]. Within adipose tissue, denser tissue (smaller adipocytes versus larger lipid filled adipocytes) is associated with increased mortality risk in older adults [8]. Given the general consistency of tissue characteristics in the body (ie. fatty liver is positively correlated with visceral adipose and skeletal muscle adipose [2]) it is likely that the characteristics of other tissues may also be associated with health risks. 

Mammography for breast cancer screening is one of the most widely used forms of imaging but the prognostic value of mammographic images outside of breast cancer risk prediction is largely unknown. In mammography, three metrics which reflect the tissue composition of the breast have been commonly characterized in epidemiologic studies: 1) nondense area which represents breast fat tissue, 2) dense area which refers to fibroglandular tissue and stromal tissue, and 3) percent density, the amount of dense tissue relative to total breast area. Density measures can be estimated quantitatively including planimetry [9], computerized thresholding techniques [10] or qualitatively via visual assessment, for example the Breast Imaging and Data System (BI-RADS) breast composition categories which range from almost entirely fatty to extremely dense [11]. Within the breast cancer literature, numerous studies have demonstrated that greater dense area and percent density are strong risk factors for breast cancer [12-14]. Nondense area may also confer risks for breast cancer independent of breast density although the direction of the association is unclear [15,16]. Although the reasons underlying these associations are incompletely understood, mammographic density has emerged as an important factor for the prediction of breast cancer risk [17]. 

It is less clear if mammographic features relate to survival among women diagnosed with breast cancer. Several studies have reported no association between breast density and breast cancer mortality [18-21] or increased risk of breast cancer mortality [20] among breast cancer cases. A study of breast tissue density among breast cancer cases relative to cases and non-breast cancer controls combined, reported that high density breast tissue was associated with decreased mortality risk [22]. Even less is known regarding potential relationships of breast density with all-cause mortality in women without breast cancer. Improved understanding of potential relationships is important for helping to identify mechanisms which may be targeted for interventions. Hypothesized mechanisms linking breast density and breast cancer mortality such as paracrine growth factors and genetic damage [23] may be pertinent to the development of disease beyond breast cancer such as cardiovascular disease [24] and arthritis [25]. Therefore, the aim of this study is to prospectively investigate relationships between nondense and dense mammographic area, breast area and percent density with risk of all-cause mortality in women without breast cancer. 

## Methods

### Study population

Data was derived from the Breast Cancer Detection Demonstration Project (BCDDP), a study sponsored by the National Cancer Institute and the American Cancer Society. Between 1973 and 1975, 284,780 women aged 35 through 74 years were accrued. The screening phase of the study lasted from 1973 to 1979, during which time women in 29 centers in 27 cities across the United States were screened annually for incident breast cancer [26]. 22 centers sent mammographic images to the BCDDP resource center. In 1980 a subset of 59,907 without a diagnosis of breast cancer during the screening phase were selected for a long-term follow-up study (phase I, 1980-1986) and further follow-up in phase II (1987-1989) and phase III (1993-1995). Details on the study design are provided in Benichou et al. [27]. 

Mammographic measurements were completed on 7,251 women. We additionally restricted our analysis to women who were 1) also sampled in the follow-up phase, and 2) had no breast cancer diagnosis at screening or follow-up herein referred to as “healthy women”. Thus, for this analysis data are from 4,424 healthy women which includes women with benign breast disease, women recommended for biopsy and women without abnormality or breast biopsy recommendation. We focused on healthy women because the primary aim of this analysis was to determine whether mammographic measures are associated with mortality risk beyond breast cancer. Of the 4,424 healthy women, 2 women who were deceased but missing date of death were excluded. Because body mass index (BMI) is an adverse prognostic factor for breast cancer [28] and is strongly related to breast density [23], women missing BMI (N=65) were excluded. Ten women with grossly inaccurate BMI (ie. BMI>2000 kg/m^2^) and 102 underweight women (BMI <18.5kg/m^2^) were also excluded due to possible confounding effects on mortality resulting in an analytical sample of 4,245 women. 

### Ethics Statement

The BCDDP follow-up cohort study was approved by the Institutional Review Board at the National Cancer Institute. All participants provided written informed consent. 

### Vital status

Follow-up information included vital status (alive or deceased) and date of death obtained from the National Death Index through December 31, 2005. Women who were not identified as deceased were censored at the date through which vital records were complete. 

### Mammographic features

Mammographic images from the baseline screening examination of the BCDDP consisted of xerograms (N=3,474) and x-rays (N=771). Measured breast area with dense mammographic appearance was assessed quantitatively with a compensating polar planimeter (LASICO 1280-12; Los Angeles, CA) on the cranio-caudal view. Using the mammogram image, the reader used a wax pencil to outline the entire breast and the portions of breast containing radio-densities. The reader used the planimeter to trace the outlines of the entire breast and dense breast to compute total breast area and dense breast area, respectively. Nondense area was defined as the difference between total breast area and dense area. Percent density was calculated as dense area divided by the total breast area. Values reported are the average of the left and right breast or the reading of the non-missing breast if only one breast was read. These measures were previously determined and described in detail by Benichou et al. [27] and Byrne et al. [9] who reported a positive association between mammographic density and breast cancer risk among women in the BCDDP cohort. Quality control procedures demonstrated acceptable intra and inter observer measurement reliability [27]. 

### Covariates

Covariates were chosen a priori and included factors that may potentially confound relationships between mammographic features and mortality including age at mammogram, education, race, type of mammogram (xerogram or x-ray), BMI, smoking status, prevalent self-reported diabetes and heart disease. Education was categorized as less than high school, high school graduate or postsecondary. Race was self-identified and categorized as non-Hispanic Caucasian, non-Hispanic Black, Hispanic, Oriental (Japanese, Chinese, other Oriental) or other racial/ethnic group. BMI was calculated from measured height and weight during the screening phase. If multiple measures of weight and height were recorded, the measures closest to the mammogram were used. Information on age started smoking, and date of diagnosis of diabetes and/or heart disease were ascertained from questionnaires during the follow-up period. Summary variables that represent the baseline screening characteristics of women were determined by comparing the study entry date for each participant to the age at smoking onset and date of diagnosis of diabetes and/or heart disease. Smoking status was categorized as never, current, former, or unknown whether current or former. 

### Statistical analysis

Participant characteristics are presented as medians with interquartile ranges for covariates that were not normally distributed (age and BMI). To meet linearity assumptions, BMI, nondense area and breast area were log-transformed, dense area and percent density were square root transformed. Correlations between age, mammographic features and BMI were examined using the Spearman correlation coefficient (r). The distribution of nondense area, dense area, breast area and percent density were skewed towards the extremes even following transformation and were thus categorized into percentile groups that reflected their distribution in the analytic sample (15^th^, 45^th^, 60^th^, and 85^th^). Sensitivity analyses with a ± 5% change in percentile categories yielded similar results. 

Cox proportional hazards models were used to examine independent associations of nondense area, dense area, breast area and percent density with all-cause mortality. The time metric was years to death or follow-up. Risk analyses were conducted per standard deviation (SD) difference in continuous mammographic measures and with categorized mammographic measures. For categorical analysis of mammographic features, the risk of mortality in each of the upper three percentile groups was compared to the risk for the lowest group. Examination of Kaplan-Meier curves and Schoenfeld residuals indicated that the proportional hazards assumption was not violated. Risk relationships are presented in sequentially adjusted models. Tests for interactions between age and mammographic features were not significant (P>0.05) and thus models were age-adjusted and not age-stratified. We first examined unadjusted associations between mammographic measures and mortality (Model 1). Model 2 is age-adjusted. Model 3 additionally adjusted for baseline variables: education, race, smoking history, type of mammogram, prevalent diabetes and heart disease. Since mammographic features strongly reflect obesity, and obesity is a risk factor for mortality, we adjusted for BMI in a separate model (Model 4) to assess whether mammographic features were independently associated with mortality. The Wald statistic was used to test for an overall effect of categories of mammographic features on risk of mortality. 

Sensitivity analyses were conducted with additional covariates (menopause at time of mammogram and age at first live birth) that have been implicated in the pathogenesis of breast cancer and may influence breast tissue characteristics. Statistical significance was determined at P<0.05. STATA version 12.1 (StataCorp, College Station, TX) was used for all analyses. 

## Results

Table 1 presents transformed mammographic measures and additional characteristics of the analytical sample. The median (IQR) age of the analytic sample was 50 years (44-57 years). More than 94% of the analytic sample is white, and 45% have postsecondary education. Generally, participants with higher nondense area are older, less educated, have heavier BMI, and are more likely to have diabetes and heart disease (P<0.05 for all). 

**Table 1:** Characteristics of the overall analytical sample and by percentiles of nondense mammographic area.

	**Analytical sample**	**Percentiles of nondense mammographic area**				
		15	40	65	85	*P* value
No. Participants	4245	636	1486	1487	636	
Age in years, median (IQR)	50 (44-57)	45 (40-50)	49 (44-55)	52 (46-58)	54 (47-61)	<0.001
White race, n (%)	4004 (94.3)	604 (95.0)	1426 (96.0)	1398 (94.0)	576 (90.6)	<0.001
Postsecondary education, n (%)	1915 (45.1)	349 (54.9)	715 (48.1)	617 (41.5)	234 (36.8)	<0.001
BMI in kg/m^2^, median (IQR)	23.3 (21.5-25.9)	20.8 (20.0-22.1)	22.3 (21.1-23.7)	24.6 (22.8-26.6)	28.3 (25.8-31.4)	<0.001
18.5-24.9 kg/m^2^, n (%)	2879 (67.8)	617 (97.0)	1307 (88.0)	828 (55.7)	127 (20.0)	
25.0-29.9 kg/m^2^, n (%)	990 (23.3)	16 (2.52)	171 (11.5)	516 (34.7)	287 (45.1)	
>30.0.0 kg/m^2^, n (%)	376 (8.86)	3 (0.47)	8 (0.54)	143 (9.62)	222 (34.9)	
Current smoking, n (%)	221 (5.21)	51 (8.02)	87 (5.85)	64 (4.30)	19 (2.99)	<0.001
History of diabetes, n (%)	39 (0.92)	0 (0.0)	9 (0.61)	14 (0.94)	16 (2.52)	<0.001
History of heart disease, n (%)	88 (2.07)	6 (0.94)	32 (2.15)	38 (2.56)	12 (1.89)	0.007
^a^Nondense area, median (IQR)	4.07 (3.51-4.58)	2.88 (2.62-3.07)	3.70 (3.48-3.89)	4.42 (4.24-4.61)	5.08 (4.92-5.28)	<0.001
^b^Dense area, median (IQR)	4.96 (3.26-6.48)	5.89 (4.78-6.97)	5.60 (4.31-6.90)	4.42 (2.89-6.17)	2.41 (0-4.27)	<0.001
^a^Breast area, median (IQR)	4.52 (4.19-4.85)	3.97 (3.69-4.19)	4.28 (4.08-4.49)	4.69 (4.50-4.84)	5.02 (5.01-5.37)	<0.001
^b^Percent density, median (IQR)	5.68 (3.31-7.24)	8.20 (7.57-8.67)	6.69 (5.60-7.49)	4.40 (2.97-5.73)	1.80 (0-3.11)	<0.001

Comparisons between groups from ANOVA for continuous variables and Chi-Square test for categorical variables. P value from Wald statistic to test for an overall effect across percentiles of nondense mammographic area. ^a^ Log-transformed cm^2^, ^b^ Square root-transformed cm^2^ and percent. Abbreviations-BMI: body mass index, IQR: interquartile range

Table 2 depicts correlations between age, mammographic features and BMI. Age was positively correlated with nondense area (r=0.29, P<0.001), breast area (0.19, P<0.001) and BMI (r=0.12, P<0.001), and inversely correlated with dense area (r=-0.18, P<0.001) and percent density (r=-0.28, P<0.001). Nondense area was positively correlated with BMI (r=0.68, P<0.001), and breast area (r=0.83, P<0.001) and inversely correlated with dense area and percent density (r=-0.42, P<0.001 and -0.80, P<0.001, respectively). Dense area was weakly but significantly correlated with breast area (r=0.07, P<0.001) and inversely correlated with BMI (r=-0.17, P<0.001). Breast area was negatively correlated with percent density (r=-0.37, P<0.001) and positively correlated with BMI (r=0.64, P<0.001). As expected due to its derivation, percent density was positively correlated with dense area (r=0.85, P<0.001). In summary, older women had higher BMI and larger breasts characterized by greater nondense area and lower dense area. 

**Table 2:** Spearman correlations (r) of age, continuous mammographic measures and BMI^**a**^.

	Age	Nondense area	Dense area	Breast area	Percent density	BMI
Age	1.00					
Nondense area	0.29	1.00				
Dense area	-0.18	-0.42	1.00			
Breast area	0.19	0.83	0.07	1.00		
Percent density	-0.28	-0.80	0.85	-0.37	1.00	
BMI	0.12	0.68	-0.17	0.64	-0.46	1.00

a P<0.001 for all correlations. Abbreviation-BMI: body mass index

The mean (SD) follow-up period was 28.2 (4.75) years during which time 1361 women died (mortality rate 139/1000 person years). 45 women who were not known to have breast cancer during the BCDDP follow-up study died due to breast cancer. Associations between continuous mammographic features and risk of mortality per SD difference are shown in Table 3. In unadjusted models (Model 1), greater nondense area and breast area were associated with increased risk of mortality. Conversely, greater dense area and percent density were associated with lower risk of mortality. All associations were reduced but remained significant after adjustment for age (Model 2) and did not change appreciably after adjustment for additional covariates (Model 3). With additional adjustment for BMI (Model 4), associations were diminished for all features but remained significant for dense area (HR 0.94, 95% CI 0.89-0.99, P=0.02) and percent density (HR 0.93, 95% CI 0.87-0.98, P=0.01). Within models older age and greater BMI were consistently associated with increased mortality risk (P<0.001). 

**Table 3:** Associations between mammographic measures (per standard deviation difference) and risk of mortality.

	Model 1^a^ HR (95% CI)	P	Model 2^b^ HR (95% CI)	P	Model 3^c^ HR (95% CI)	P	Model 4^d^ HR (95% CI)	P
Nondense area	1.47 (1.39-1.55)	<0.001	1.17 (1.10-1.24)	<0.001	1.16 (1.09-1.24)	<0.001	1.07 (0.99-1.15)	0.10
Dense area	0.80 (0.76-0.84)	<0.001	0.91 (0.86-0.95)	<0.001	0.91 (0.86-0.96)	<0.001	0.94 (0.89-0.99)	0.02
Breast area	1.32 (1.25-1.39)	<0.001	1.13 (1.06-1.19)	<0.001	1.12 (1.06-1.18)	<0.001	1.02 (0.95-1.09)	0.67
Percent density	0.73 (0.70-0.77)	<0.001	0.87 (0.83-0.92)	<0.001	0.88 (0.83-0.93)	<0.001	0.93 (0.87-0.98)	0.01

^a^ Model 1 unadjusted; ^b^ Model 2 age-adjusted; ^c^ Model 3 adjusted for age, education, race, smoking history, type of mammogram (xerogram versus x-ray), diabetes and heart disease. ^d^ Model 4 adjusted for Model 2 covariates plus body mass index. P value from Wald statistic. All statistical tests were two-sided. Abbreviations-CI: confidence interval, HR: hazard ratio.

Associations between percentiles of mammographic features and risk of death yielded similar results (Table 4). Compared with women in the 15^th^ percentile, the upper three groups of nondense area and breast area had increased risk of mortality (Model 1). Associations were reduced with adjustment for age (Model 2) and but remained significant for the upper 2 groups of nondense area and the upper 3 groups of breast area (Model 3). Inverse relationships were observed for dense area and percent density; compared with the 15th percentile, women in the upper three groups had a lower risk of mortality (Model 1) that persisted after adjustment for age and additional covariates (Models 2 and 3). Adjustment for BMI (Model 4) attenuated associations except for marginal associations with the upper group of dense area (HR=0.83, 95% CI 0.68-1.00), and the upper groups of percent density: 40^th^ percentile (HR=0.86, 95% CI 0.74-0.99), 65^th^ percentile (HR=0.84, 95% CI 0.71-0.996) and 85^th^ percentile (HR=0.80, 95% CI 0.64-1.00). Results were similar when models of percentiles and continuous mammographic features were adjusted for menopause status and age at first birth. 

**Table 4:** Associations between percentiles of mammographic measures and risk of mortality.

Percentiles	No. of deaths	No. person yrs	Rate	Model 1^a^ HR (95% CI)	P	Model 2^b^ HR (95% CI)	P	Model 3^c^ HR (95% CI)	P	Model 4^d^ HR (95% CI)	P
Nondense area											
15	120	1567	76.6	1.00	<0.001	1.00	<0.001	1.00	<0.001	1.00	0.23
40	406	3566	114	1.53 (1.25-1.88)		0.99 (0.80-1.21)		1.04 (0.84-1.28)		0.99 (0.80-1.22)	
65	555	3443	161	2.28 (1.87-2.78)		1.18 (0.96-1.44)		1.23 (1.01-1.52)		1.08 (0.87-1.35)	
85	308	1413	218	3.29 (2.66-4.06)		1.44 (1.16-1.80)		1.53 (1.23-1.91)		1.22 (0.94-1.58)	
Dense area											
15	273	1440	189	1.00	<0.001	1.00	0.006	1.00	0.04	1.00	0.25
40	521	3469	150	0.76 (0.66-0.88)		0.88 (0.76-1.02)		0.83 (0.72-0.97)		0.92 (0.79-1.08)	
65	422	3548	119	0.59 (0.50-0.68)		0.77 (0.66-0.90)		0.82 (0.70-0.95)		0.92 (0.78-1.08)	
85	173	1534	113	0.55 (0.45-0.66)		0.79 (0.65-0.95)		0.77 (0.63-0.93)		0.83 (0.68-1.00)	
Breast area											
15	132	1558	84.8	1.00	<0.001	1.00	0.001	1.00	0.003	1.00	0.39
40	457	3528	130	1.59 (1.31-1.93)		1.19 (0.98-1.45)		1.26 (1.04-1.54)		1.17 (0.96-1.43)	
65	513	3472	148	1.85 (1.52-2.24)		1.22 (1.01-1.48)		1.30 (1.07-1.58)		1.10 (0.89-1.35)	
85	287	1434	200	2.66 (2.17-3.27)		1.51 (1.23-1.86)		1.51 (1.22-1.88)		1.12 (0.87-1.44)	
Percent density											
15	284	1430	199	1.00	<0.001	1.00	<0.001	1.00	0.001	1.00	0.13
40	563	3430	164	0.79 (0.69-0.92)		0.83 (0.72-0.96)		0.79 (0.68-0.91)		0.86 (0.74-0.99)	
65	412	3571	115	0.53 (0.46-0.62)		0.71 (0.61-0.83)		0.74 (0.63-0.86)		0.84 (0.71-0.996)	
85	130	1560	83.3	0.37 (0.30-0.45)		0.71 (0.57-0.87)		0.68 (0.55-0.84)		0.80 (0.64-1.00)	

^a^ Model 1 unadjusted; ^b^ Model 2 age-adjusted; ^c^ Model 3 adjusted for age, education, race, smoking history, type of mammogram (xerogram versus x-ray), diabetes and heart disease. ^d^ Model 4 adjusted for Model 2 covariates plus body mass index. P value from Wald statistic to test for an overall effect of categories of mammographic measures. All statistical tests were two-sided. Abbreviations-CI: confidence interval, HR: hazard ratio.

## Discussion

This study contributes to our understanding of the prognostic value of mammographic features by examining associations with mortality risk in healthy women, a seldom studied area. Greater nondense area and total breast area were associated with increased mortality risk but associations were not independent of BMI. In contrast, greater dense area and percent density were both associated with lower mortality risk independent of risk factors and BMI. For every SD increase of dense area, there was a 6% reduction in mortality risk and for every SD increase of percent density, there was a 7% reduction in mortality risk. These results suggest that mammographic images may capture features that are independently related to survival. Thus, when evaluating mammograms in relation to breast cancer risk, the characteristics of breast tissue and specifically, dense tissue and percent density may provide novel prognostic information for healthy women. 

In this population the association between mammographic features and risk of death appears to largely reflect risk carried by overweight or obesity. All mammographic features were correlated with BMI (nondense area and breast area positively, dense area and percent density inversely). Our risk models also seem to reflect the risk for mortality attributable to excess body weight [29-31]. The upper percentile groups of nondense area and total breast area that carried the greatest mortality risk in unadjusted analyses predominately consisted of women who were overweight or obese. Moreover, adjustment for BMI reduced associations and significantly attenuated associations with nondense area and breast area. However, inverse associations remained statistically significant for continuous measures of dense area and percent density after adjustment for BMI. This suggests that associations between mammographic features and mortality risk may extend beyond risk attributable to BMI alone. 

The basis for the observed associations between mammographic density characteristics and mortality risk are unclear and likely complex. Relationships between adipose tissue, disease and disease-related mortality are possibly mediated through carbohydrate metabolizing characteristics of adipose tissue, secretion of cytokines [32] and/or adipokines [33]. Thus the metabolic characteristics of adipose tissue may contribute to our finding of greater mortality risk with greater nondense fatty tissue and help explain why associations were attenuated with adjustment for overall adiposity (BMI). Proposed mechanisms linking mammographic density and breast cancer risk have included steroid sex hormones and growth factors since they are generally positively associated with breast density and are involved in the development of breast cancer [23]. These stimuli also contribute to the pathogenesis of multiple diseases that impact overall life expectancy [24,25,34]. However, our data raises questions regarding this hypothesis as we observed an inverse association between dense area, mammographic density and risk of death. Alternatively, genetic factors may play a role in risk relationships. The variation in breast density attributed to lifestyle factors including hormone levels, age, age at parity, and BMI has been estimated to be only 20 to 30% [35] with the remainder possibly attributable to genetic factors [36]. As genes involved in breast density are identified, our understanding of associations between breast density and mortality risk may be expanded. 

Direct comparison of our results is limited by the lack of studies on mammographic features and health outcomes in healthy women but parallels can be drawn to studies of breast cancer mortality. The association between greater dense area and lower mortality we observed is consistent with a study of breast cancer cases from a Danish screening study that reported lower risk of all-cause mortality in women with “mixed/dense” breasts [22]. The positive association we found between nondense fat tissue and mortality risk (although not independent of BMI) is consistent with a study of breast cancer cases in which Gierach et al. [18] reported increased risk of breast cancer mortality in obese women with BI-RADS 1 density (almost entirely fat). In contrast, Chiu et al. [37] reported significant increased mortality from breast cancer among women with greater dense tissue, however, breast tissue was broadly categorized as “dense” or “nondense”. Our results vary from studies of breast cancer cases that found null associations between breast density and mortality, however these studies incompletely adjusted for confounders of prognosis such as comorbidities, treatment, tumor size or stage of cancer [19,20,38]. 

It is likely that the associations we observed with mammographic features and mortality risk are not specific to a body compartment or tissue, but rather represent a systemic effect. For example, obesity manifests as fatty infiltration of the liver [2] and skeletal muscle [39], larger adipocytes in adipose tissue [8] and as in this study, greater breast fat tissue. To that end, our results are consistent with studies of computed tomography density that show non-breast tissue density provides important prognostic information related to health outcomes [2,6,40]. Specifically, lower density skeletal muscle based on the Hounsfield Unit, has been associated with cancer-related mortality [41] and more dense adipose tissue is a risk factor for all-cause mortality in healthy older adults [8]. Although the measurement of tissue “density” differs across studies, together these findings suggest consistent associations between radiographic characteristics of tissue and mortality. Studies with whole body imaging would improve our understanding of the underlying biological characteristics of tissue density throughout the body. 

Strengths of this study include a long follow-up period of more than 30 years and the quantitative continuous measures of breast nondense and dense areas. Continuous measures overcome the limitations of BI-RADS classification which can have substantial interobserver variability [42], potentially resulting in misclassification. A limitation of this analysis concerns the design of the BCDDP cohort which was originally conceived as a general population study to demonstrate the feasibility of large-scale breast cancer screening. As a result, data on smoking, heart disease and diabetes were not collected at the time of the screening mammogram. Status was based upon recalled history in follow-up questionnaires possibly leading to misreporting. There has also been a shift towards heavier body weights since this study was initiated [43] which may limit the generalization of our results to contemporary populations. Studies in breast cancer suggest that associations between mammographic measures and mortality risk are greatest among women with BMI >30kg/m^2^ [44] and it is possible that we did not fully capture the risk relationships. The distribution of body weight in the population also limited our ability to stratify risk analyses by BMI. Given the strong attenuating effect of BMI on associations between mammographic features and mortality, there is the potential for residual confounding. 

It is also important to acknowledge potential bias within our participant population when considering the generalizability of our results. Our sample of healthy women included women with benign breast disease and women recommended for biopsy at baseline screening, who may have had a higher risk of breast cancer than the general population. However, we excluded women with incident breast cancer during follow-up screening and only a small number of women in our population (N=45) died from breast cancer. It is likely that our analytical sample is healthier than the general population. The prevalence of smoking is low, and the prevalence of diabetes is lower than women in the Second National Health and Nutrition Examination Survey which was conducted during a similar time period as the BCDDP [45]. Additional analyses in large populations to see whether our results replicate would be valuable. 

## Conclusions

Mammographic images are often not exploited beyond assessment of breast abnormalities and breast cancer risk but as we demonstrate here tissue characteristics captured with mammography may carry broader health risks. There are potential public health implications from our results given the availability of mammographic images from widespread annual mammographic screening. Further studies are needed to replicate these results and explore potential mechanisms. Although these questions require clarification, the results of this study suggest a secondary role for analyses of mammographic images that may impact estimation of mortality risk in healthy women. 
